# Involuntary Intoxication from a Medico-Legal Standpoint1Read before the Alumni Association of Niagara University, May 12,1898.

**Published:** 1896-09

**Authors:** Sydney A. Dunham

**Affiliations:** Buffalo. N. Y.; Adjunct professor of-physiology, Medical Department Niagara University, 180 West Chippewa Street


### INVOLUNTARY INTOXICATION FROM A MEDICO-LEGAL STANDPOINT.[1]

By SYDNEY A. DUNHAM, M. D., Buffalo, N. Y.,
Adjunct professor of physiology, Medical Department Niagara University.

ALCOHOLIC excesses, when once established, may be classed under three divisions, as follows: voluntary drinking, producing voluntary intoxication; voluntary drinking, producing involuntary intoxication; involuntary drinking, producing involtary intoxication. The third division of drunkenness follows the second, and the second the first, unless arrested by heroic medical and legal interference, the same as in the three stages of consumption, where the first rapidly leads to the other two. Under our first division of alcoholics, we find drunkenness more of a vice than a disease, and it should be treated with severe penalties. This class seem to drink for the fun of it, and have a desire to get drunk. When any interference is offered, the reply is that they have a personal right to do as they choose. Such people never stop to think that those living in the same neighborhood and community have personal rights as well.

Under the second division we may class those men and women who drink socially, with no intention of ever becoming under the influence of liquor. A certain proportion of these people, who should soon come to have reason dethroned by the toxic effect of alcohol, on account of the remorse and chagrin that follow, are kept from future drinking and thereafter drunkenness.

In the third division we find men and women who frequently have an uncontrollable desire to take a drink. There is an insane impulse and mania for liquor, which controls the person; the thoughts and feelings are disordered and the power of volition is suspended. Among this class of patients, who properly come under the term dipsomaniacs, the question of medical and legal responsibility is quite unsettled. With these victims the mania is increased with the first drink, the will weakened, and the unexpected, involuntary drunk follows.

The alcoholic is receiving a fair consideration at the hands of medicine, but from a legal standpoint his treatment is not so fair and just, yet apparently necessary.

While medical opinions are said to be given often with a hole in them, the law in regard to drunkenness has apparently a double

1. Read before the Alumni Association of Niagara University, May 12, 1896.

advantage, and the victim may easily escape or be unjustly punished. Different countries have different penalties for drunkenness. The penalties for crimes committed while drunk vary, because there is a difference of opinion as regards their responsibility. It is said that the laws of Greece were such that he who committed crime while intoxicated should receive a double punishment—one for the crime, the other for getting drunk. In the Roman laws we find drunkenness mitigating the penalty for the crime. In Sweden a divorce is granted for inebriety. In Italy the criminal who is intoxicated gets one-third off the sentence. In England and Scotland, a man who voluntarily becomes under the influence of liquor and commits a crime, although not thought of when sober, is considered responsible. In our state we find the following laws :

Section 21, Penal Code, State of New York :

A person is not excused from criminal liability as an idiot, imbecile, lunatic or insane person, except upon proof that, at the time of committing the alleged criminal act, he was laboring under such a defect of reason as either not to know the nature and quality of the act he was doing, or that the act was wrong.

Our courts have decided, People v. Sprague, 2 Park., 43 :

*Insane Impulses.*—If the act complained of was done under an insane impulse, which at the time destroyed the capacity of distinguishing between right and wrong, the party is not responsible.

Section 22, Penal Code, State of New York :

No act committed by a person while in a state of voluntary intoxication shall be deemed less criminal by reason of his having been in such condition. But whenever actual existence of any particular purpose, motive or intent is a necessary element to constitute a particular species or degree of crime, the jury may take into consideration the fact that the accused was intoxicated at the time in determining the purpose, motive or intent with which he committed the act.

Supreme Courts of New York State have decided under this section :

(*a*) Voluntary intoxication, though amounting to phrensy, is no defense where a homicide is committed without provocation.

(*b*) Voluntary intoxication is no excuse for crime, but the proof of it may reduce the crime of murder to the second degree, by showing absence of deliberation.

(*c*) Where intoxication results in a fixed mental disease of some continuation and duration it will relieve from criminal responsibility.

(*f*) Delirium tremens, like insanity, if it deprive one of the capacity of knowing right from wrong, saves him from any criminal responsibility for his acts.

Section 200, Penal Code, State of New York:

> A physician or surgeon, or person practising as such, who being in a state of intoxication, without a design to effect death, administers a poisonous drug or medicine, or does any other act as a physician or surgeon, to another person, which produces the death of the latter, is guilty of manslaughter in the second degree.

A doctor, merchant, lawyer or priest who is suffering from alcoholic hallucinations, and does not know a bed-post from a rattlesnake, certainly would not know the difference between morphine and baking soda, or carbolic acid and camphorated oil. In this section the physician is held responsible for getting intoxicated, as the question of irresponsibility at the time of committing the act is not apparently considered.

The law says much about voluntary intoxication and little about involuntary intoxication. The great question to be decided and proved is, whether the intoxication was *voluntary* or *involuntary*. Under the third division of alcoholism, that of involuntary intoxication, there are many victims.

Inebriety gradually passes from the voluntary periods of drink to the involuntary. It is impossible for an inebriate to become voluntarily intoxicated when he has repeatedly shown that he had no will power and was beyond self-control. The responsibility of dipsomania must begin when the intoxications are voluntary, and here alone is the proper place for punishment; and it should be so severe as to prevent the victim from developing the involuntary stages. Our voluntary drinkers, who are responsible for their voluntary intoxications of today, will, by and by, commit heinous crimes in a state of involuntary intoxication, for which they will be irresponsible. The law would not hold a patient responsible for an act committed under ether or chloroform narcosis. Acts from the anesthetised brain of the alcoholic are insane impulses as well. Experience in studying these cases, and from answers given to a uniform set of questions, leads me to the belief that there are many criminals punished for crimes committed while intoxicated, who were irresponsible for their acts. These are the questions asked men who have been in the habit of getting drunk, but when sober are able, honest and reputable persons :

Q. 1. After intoxication, were you able to recall what occurred while in that state? A. Very seldom, if ever, could I recall anything I did.

Q. 2. Did you ever have a tendency to suicide? A. Yes.

Q. 3. Would you think it just to hold you responsible for acts committed while you were in the midst of a drinking period? A. Not at all. Any more than to hold a person of unsound mind responsible.

A legal gentleman gives this answer to question three : No, not just ; but, perhaps, necessary to the well-being of society. To question one, the majority of the answers of older drinkers was in the negative. To question two, about half of the answers were in the affirmative. To question three, all replied that it would be unjust.

Many alcoholic patients can be properly classed under Horatio C. Wood's definition of insanity : "Insanity is a condition of mental aberration sufficiently intense to overthrow the normal relations of the individual to his own thoughts and acts, so that he is no longer able to control them through his will."

Those who have continued to excess for three years the periodical or constant drinking of alcoholics, are frequently irresponsible for acts committed while under the influence of liquor, as they do not voluntarily become intoxicated, because they have fixed mental disease and because they have insane impulses. A person when intoxicated does things which would be attempted only by insane individuals. Their hallucinations and delusions are as fixed for a time and as completely control them as those which are common to persons mentally deranged from brain lesions. During this period of mental aberration, acts are committed without any forethought or consciousness on their part. They know no more what occurs or what they do than an epileptic during an attack. A mother worships her child, yet drowns it when intoxicated. A husband loves his wife, yet beats her into insensibility when drunk. A son, whom his mother had supported from childhood by days' work, while in the midst of a drinking period, goes home and knocks her brains out. A father kisses his little ones every morning and promises them bread and butter on Saturday when he gets his week's wages; when Saturday comes, gets drunk on his money, reaches home on Monday without a cent, but in time to drink the milk sent in by kind neighbors to a starving infant. There is no question about the insanity of the person under these circumstances and a thousand more similar ones that might be given. They are irresponsible from a legal standpoint, because they are insane and had no idea of right or wrong when the act was committed. According to law, many such persons have paid the penalty for a crime committed, which they knew nothing about, although they were considered irresponsible at the time, yet they were held responsible for the crime of getting drunk. If getting under the influence of liquor entails such responsibility, the law should enforce a more severe penalty for drunkenness and thereby give society another lift toward preventing it.

A well-known gentleman, after a debauch, always inquired of his friends if he had done anything bad, as he was always worried until he was assured he had done no harm. Frequently the police reports give accounts of some alcoholic who claims to have been the victim of highway robbery, which, after investigation, proved to be without foundation. A drunkard's hallucinations and delusions are of the most dangerous kind—dangerous to himself as well as to others. The danger of self-destruction while under the influence of liquor is manifested in their over-estimated strength and ability to act, wherein they drown because they thought they could swim ; or are killed because they had no fear of the locomotive ; or commit suicide to keep from falling into the hands of imaginary pursuers ; or by jumping from windows when the furniture of the room begins to turn over and is transformed into processions of animals, such as elephants, monkeys and snakes. They are dangerous to others because, when intoxicated, their worst natures have control. Delusions of infidelity in the home circle are present, and by their hallucinations they kill their best friends in trying to escape from imaginary enemies, robbers and particularly the devil.

When a man knowingly incapacitates himself for self-government he is responsible. This the drinker does voluntarily with his first few years of debauches. After this the majority of drinkers involuntarily become intoxicated. The community should allow no man, voluntarily or involuntarily, to enter into a hibernating period and lose all control of his thoughts, words and actions. The lower animals take some means of protection during their seclusion at such times ; but the drunkard never, because his natural instincts are benumbed, saying nothing of his intelligence.

When punishment fails to stop a man from drinking liquors or taking any stimulus to excess, the man should be considered as one who has delusions which control him, and held for treatment as one who at times is excitable, dangerous and destructive. Surely the periodic drinker is irresponsible enough to debar him from any responsible position. There is no price large enough to keep such people from taking liquor if it can be had. Family and social ties, position, and honor and fortune, are often sacrificed in satisfying the morbid desire for drink. When an individual is controlled by a delusion, an illusion or hallucination, followed by an insane impulse, if it be only for a few minutes, he is as much irresponsible for things done in that time as though they had lasted several days or months. In *petit mal*, one form of epilepsy, a person has complete loss of consciousness for only a few seconds.

Many drinkers give a history of mental aberration of a few seconds in duration, and express themselves as having observed that there were times when they would find themselves staring at something without any idea as to time. These unfortunates can seldom tell why they take the first drink. Repeated experiences with them have proven beyond a doubt that a drink means a drunk. Previous to and during the drinking period, there seems to be an aura with them similar to that of the diseased epileptic, wherein there is partial or complete loss of consciousness, with mental weakness.

Alcoholism predisposes to epilepsy, and certain forms of *petit mal* seem to predispose to excesses in drink. There are in every community men and women whose better judgment, although weakened mentally and morally, saves them from gratifying morbid appetites and violating the law. In these cases the little judgment is at a premium with them, for as soon as they begin to take liquor they rapidly lose the good-will power they previously possessed, and their impulses become uncontrollable, and he who would commit no greater offense than a misdemeanor without liquor would commit murder by using it.

An intermediate place is required, between the state asylum and the penitentiary, for the inebriate. Here is a case for example: A young man becomes intoxicated and during the drinking period steals several articles which he throws away or he pawns for money. He is honest when not drinking; had never stolen until he began the use of liquor. He repeatedly gets drunk and steals through insane impulses and finally is arrested, and the law says he shall pay the penalty for larceny by going to the penitentiary. The doctors find it difficult to make out papers of lunacy and the asylums do not care to keep him after he regains complete consciousness, which is usually after a few days. Nothing but the law will restrain these cases for a time necessary for treatment to do them good. Because persons commit crime while under involuntary intoxication it is unjust to say they are criminals, and because they are temporarily unbalanced by drink it does not prove them lunatics. It does give them a clear history of a diseased condition of the body and mind, and should incapacitate them for personal liberty until proper treatment has been administered.

180 West Chippewa Street.

